# Stress time-dependently influences the acquisition and retrieval of unrelated information by producing a memory of its own

**DOI:** 10.3389/fpsyg.2015.00910

**Published:** 2015-06-30

**Authors:** Chelsea E. Cadle, Phillip R. Zoladz

**Affiliations:** Department of Psychology, Sociology, and Criminal Justice, Ohio Northern University, Ada, OHUSA

**Keywords:** stress, long-term potentiation (LTP), hippocampus, amygdala, metaplasticity

## Abstract

Stress induces several temporally guided “waves” of psychobiological responses that differentially influence learning and memory. One way to understand how the temporal dynamics of stress influence these cognitive processes is to consider stress, itself, as a learning experience that influences additional learning and memory. Indeed, research has shown that stress results in electrophysiological and biochemical activity that is remarkably similar to the activity observed as a result of learning. In this review, we will present the idea that when a stressful episode immediately precedes or follows learning, such learning is enhanced because the learned information becomes a part of the stress context and is tagged by the emotional memory being formed. In contrast, when a stressful episode is temporally separated from learning or is experienced prior to retrieval, such learning or memory is impaired because the learning or memory is experienced outside the context of the stress episode or subsequent to a saturation of synaptic plasticity, which renders the retrieval of information improbable. The temporal dynamics of emotional memory formation, along with the neurobiological correlates of the stress response, are discussed to support these hypotheses.

## What is Stress?

Stress is experienced during situations that pose a threat to an organism and leads to the activation of two major physiological systems, the sympathetic nervous system (SNS) and the hypothalamus-pituitary-adrenal (HPA) axis. SNS activation allows for the immediate fight-or-flight response through rapid release of epinephrine (EPI) and norepinephrine (NE) from the adrenal medulla (Gunnar and Quevedo, 2007). Activation of the HPA axis, on the other hand, leads to a slower response, eventually resulting in the release of corticosteroids from the adrenal cortex (de Kloet et al., 1999; Joels, 2001).

Stress response neurochemicals exert a profound effect on learning and memory by influencing cognitive brain areas, such as the hippocampus, prefrontal cortex (PFC), and amygdala. Both the hippocampus, which is crucial for the formation of declarative and spatial memories (Moser and Moser, 1998; Kaut and Bunsey, 2001; Broadbent et al., 2004; Eichenbaum, 2004; Squire et al., 2004; Broadbent et al., 2006), and the PFC, which is responsible for working memory and higher-order cognitive function (Rowe et al., 2001; Bechara, 2005; Nebel et al., 2005; Muller and Knight, 2006), have a high density of corticosteroid receptors (McEwen et al., 1968, 1969; Mcewen et al., 1970; Diorio et al., 1993; McGaugh, 2004), making them highly susceptible to the effects of stress. The amygdala is primarily responsible for the processing of emotional information and serves to exacerbate the stress response by enhancing HPA axis activity (McGaugh, 2004; Roozendaal et al., 2009). Stress differentially impacts learning that is dependent on these brain areas, and when considering the different forms of stress-memory interactions, the most complex appears to be that of stress effects on hippocampus-dependent memory (Zoladz et al., 2014a), which will be the focus of this review.

## Type of stress

Stress effects on learning and memory depend on the type of stressor that is employed. Intrinsic stress is a stressor that is intrinsic to, or a part of, the learning experience, and extrinsic stress is a stressor that is extrinsic to, or outside, the learning experience. In general, intrinsic stress (e.g., emotionally arousing words in a word list, colder water temperature in a Morris water maze) facilitates learning and memory (Sandi et al., 1997; Cahill and McGaugh, 1998). Extrinsic stress (e.g., exposing participants to a stressor and then having them learn a word list, shocking rats and subsequently testing their ability to navigate a maze) effects on learning and memory, on the other hand, are much more complex and can involve enhancement, impairment or no effects on cognition (Joels et al., 2006; Zoladz et al., 2011b, 2014a). In the present review, we will focus on the influence of extrinsic stress on learning and memory. During a stressful, or even traumatic, event (e.g., wartime combat, witnessing a crime), learning that occurs often results in a powerful memory for the stressor, although this may depend on what aspects (i.e., central or peripheral details) about the stressor are tested (see section below). Here, it is our goal to discuss how the physiological changes that occur during the stress impact learning and memory for events that occur subsequent to/prior to stress exposure.

## Stress Effects on Learning and Memory Depend on Stage

Learning and memory can generally be divided into three major stages: encoding, consolidation and retrieval. Encoding involves the acquisition phase, during which information is initially learned. Consolidation is when the learned information is stored in order to be successfully retrieved (the third stage) at a later point in time. Most research, in both humans and rodents, has reported facilitative effects of post-learning stress or corticosteroid administration on long-term memory consolidation (Cahill et al., 2003; Beckner et al., 2006; Hui et al., 2006; Smeets et al., 2008; Preuss and Wolf, 2009) and deleterious effects of stress or corticosteroid administration on long-term memory retrieval (de Quervain et al., 1998; Buss et al., 2004; Kuhlmann et al., 2005a,b; Buchanan et al., 2006; Diamond et al., 2006; Buchanan and Tranel, 2008; Park et al., 2008; Smeets et al., 2008; Tollenaar et al., 2008). The effects of pre-learning stress or corticosteroid administration on encoding have been more inconsistent, with studies revealing long-term memory enhancements, impairments or no effects at all (Kim et al., 2001, 2005; Jelicic et al., 2004; Elzinga et al., 2005; Diamond et al., 2006; Payne et al., 2006, 2007; Nater et al., 2007; Park et al., 2008; Schwabe et al., 2008; Duncko et al., 2009; Zoladz et al., 2011a, 2013, 2014b). Importantly, because it is administered prior to encoding, pre-learning stress can affect both the acquisition and storage of information; thus, researchers often assess short-term memory in such studies to infer what stage of information processing is being affected. A representative summary of the research studies that have examined stress effects on hippocampus-dependent learning and memory is illustrated in **Table 1**.

**Table 1:** Summary of the findings from studies examining acute stress effects on hippocampus-dependent learning and memory.

Study	Stress	Stress Timing	Stress Duration	Task	Effects	Caveats
Beckner et al. (2006)	Post-learning TSST Pre-retrieval TSST	30-min delay	10–15 min	Film	↑ LTM ---- LTM	
Buchanan and Tranel (2008)	Pre-retrieval TSST	10-min delay	20 min	Picture learning	↓ LTM	Impairment in cortisol responders
Buchanan et al. (2006)	Post-learning/pre-retrieval CPT	1-h post-learning, 10-min pre-retrieval	~3 min	Word learning	↓ LTM	Impairment in cortisol responders
Cahill et al. (2003)	Post-learning CPT	Immediately	~3 min	Picture learning	↑ LTM	
Campbell et al. (2008)*	Pre-retrieval cat exposure	Immediately or 60-min delay	~20–30 min	Water maze	↓ STM	
Conboy et al. (2009)*	Pre-retrieval/post-learning cat exposure	Immediately	30 min	Water maze	↓ STM	
Conrad et al. (2004)*	Pre-learning restraint stress	1-h delay	1 h	Y-maze	↓ STM	Only males impaired
de Quervain et al. (1998)*	Pre-retrieval foot shock	30-min delay	~1 min	Water maze	↓ LTM	
Diamond et al. (1996)*	Pre-retrieval/post-learning novelty stress	Immediately	4 h	Radial arm maze	↓ STM	Reference memory unaffected
Diamond et al. (1999)*	Pre-retrieval/post-learning cat exposure	Immediately	30 min	Water maze	↓ STM	Easy task unaffected
Diamond et al. (2006)*	Pre-learning and pre-retrieval cat exposure	Immediately	30 min	Water maze	↓ LTM	
Diamond et al. (2007)*	Pre-learning cat exposure	Immediately 30 min delay	2 min	Water maze	↑ LTM ---- LTM	
Elzinga et al. (2005)	Pre-learning cognitive stress	10–15-min delay	20 min	Word list, paragraph, spatial	↓ LTM	
Felmingham et al. (2012)	Post-learning CPT	Immediately	3 min	Picture learning	↑ LTM	Only for emotional info in females
Jelicic et al. (2004)	Pre-learning TSST	Immediately	20 min	Word learning	↓ STM ↑ STM	Impaired neutral, enhanced emotional
Kim et al. (2005)*	Pre-learning restraint + tailshock	Delayed	60 min	Water maze	↓ LTM	
Kim et al. (2001)*	Pre-learning restraint + tailshock	30–60 min delay	60 min	Water maze	↓ LTM	
Kuhlmann et al. (2005b)	Pre-retrieval TSST	10-min delay	~10 min	Word learning	↓ LTM	Only emotional words impaired
Li et al. (2013)	Post-learning/pre-retrieval TSST	1-h post-learning, immediately pre-retrieval	~15 min	Face learning	↓ STM	
Mccullough and Yonelinas (2013)	Post-learning CPT	20-min delay	3 min	Picture learning	↑ STM	
Nater et al. (2007)	Pre-learning TSST	Immediately	15–20 min	Word learning	↑ STM	Only enhanced cortisol responders
Park et al. (2006)*	Pre-retrieval/post-learning cat exposure	Immediately	30 min	Water maze	↓ LTM	
Park et al. (2008)*	Pre-learning and pre-retrieval cat exposure	Immediately	30 min	Water maze	↓ LTM	
Payne et al. (2007)	Pre-learning TSST	Immediately	20 min	Picture learning	↑ LTM ↓ LTM	Enhanced emotional, impaired neutral
Payne et al. (2006)	Pre-learning TSST	A few minutes delay	20 min	Picture learning	↓ LTM	Only impaired neutral
Payne et al. (2002)	Pre-learning TSST	Immediately	10–15 min	False memory production	↑ false memory	
Preuss and Wolf (2009)	Post-learning TSST	5-min delay	15 min	Word learning	↑ LTM	Only enhanced neutral
Quaedflieg et al. (2013)	Pre-learning MAST	Immediately 30-min delay	~10 min	Picture learning	↓ LTM	In immediate, cortisol positively associated w/recall; in delay, cortisol negatively associated w/recall
Sandi et al. (2005)*	Post-learning/pre-retrieval cat exposure	Immediately	30 min	Water maze	↑ LTM	
Schoofs and Wolf (2009)	Pre-retrieval TSST	10-min delay	15 min	Word learning	---- LTM	Only tested women in luteal phase
Schwabe and Wolf (2014)	Pre-retrieval CPT	Immediately 25-min delay 90-min delay	3 min	Word learning	---- LTM ↓ LTM ↓ LTM	
Schwabe et al. (2009)	Pre-retrieval CPT	30-min delay	3 min	Word learning	↑ LTM	Only enhanced emotional
Schwabe et al. (2008)	Pre-learning CPT	10-min delay	3 min	Word learning	↑ LTM	Only enhanced neutral
Smeets (2011)	Pre-retrieval CPT	15-min delay	3 min	Word learning	↓ LTM	
Smeets et al. (2008)	Pre-learning CPT Post-learning CPT Pre-retrieval CPT	5-min delay 5-min delay 8-min delay	3 min	Word learning	---- LTM ↑ LTM ↓ LTM	
Smeets et al. (2009)	Pre-learning TSST Pre-learning TSST Post-learning TSST	5 min delay 2 h delay 1 h delay	20 min	Stressor-related and stress-unrelated words	↑ LTM ↑ LTM ---- LTM	Only stress-related words affected
Woodson et al. (2003)*	Post-learning/pre-retrieval cat exposure	Immediately	30–45 min	Water maze	↓ STM	
Zoladz et al. (2014a)	Pre-retrieval CPT	Immediately	3 min	Word learning	↑ LTM ↓ LTM	Enhanced male cortisol responders; impaired male cortisol non-responders
Zoladz et al. (2014b)	Pre-learning CPT	Immediately	3 min	False memory production	↑ True memory ↓ False memory	Enhanced true memory in females only
Zoladz et al. (2014c)	Pre-learning CPT	Immediately	3 min	Word list learning	↑ LTM	Enhanced HR responders only
Zoladz et al. (2011a)	Pre-learning CPT	Immediately 30-min delay	3 min	Word list learning	↑ LTM ↓ LTM	Only emotional words affected
Zoladz et al. (2013)	Pre-learning CPT	30-min delay	3 min	Word list learning	↓ LTM	Only impaired in male cortisol responders
Zoladz et al. (2010)*	Post-learning/pre-retrieval IA training or IA retrieval	Immediately	30 min	Water maze	↓ STM	

CPT, cold pressor test; HR, heart rate; IA, inhibitory avoidance; LTM, long-term memory (≥24 h); MAST, Maastricht Acute Stress Test; STM, short-term memory (<24 h); TSST, Trier Social Stress Test. In some cases, the CPT was the socially evaluated version (SECPT); rat studies are marked with an asterisk (*).

The effects of stress on different stages of learning and memory appear to depend on an interaction between corticosteroid and noradrenergic mechanisms in the amygdala and hippocampus. Inactivation or lesions of the basolateral amygdala (BLA), as well as systemic or intra-BLA/intra-hippocampus administration of β-adrenergic receptor antagonists, have been shown to prevent stress and corticosteroid effects on learning and memory (Kim et al., 2001; Roozendaal et al., 2003, 2004; Kim et al., 2005; Zoladz et al., 2011b). Additionally, the effects of stress are frequently selective for emotionally arousing (i.e., amygdala-activating) information (Kuhlmann et al., 2005b; Buchanan et al., 2006; Smeets et al., 2008), emphasizing amygdala involvement in the effects. Another contributing factor to stress-memory interactions is the type of information affected. That is to say, stress often exerts differential effects on learning and memory for central and peripheral details. During stress or arousal, attention is narrowed (Easterbrook, 1959), which can hinder one’s ability to subsequently learn or remember peripheral aspects of an event or scene. Thus, in some instances, stress can enhance one’s memory for the gist, or central aspects, while impairing an individual’s ability to recollect finer details (Kensinger, 2004). These findings resonate with additional work showing that stress sometimes facilitates memory for emotional, potentially more important, information, at the cost of memory for neutral, potentially less important, information (Payne et al., 2006, 2007).

## Theoretical Approaches to Stress Effects on Cognition

Over the past several decades, numerous theories have been proposed to account for stress effects on learning and memory. Initially, researchers emphasized the deleterious effects of elevated corticosteroid levels on synaptic plasticity and related them to the effects of stress on learning (Joels and Vreugdenhil, 1998; Conrad et al., 1999). Glucocorticoid receptors (GRs), which have a lower affinity for corticosteroids than mineralocorticoid receptors (MRs), generally only become occupied when corticosteroid levels rise, such as during times of stress. The idea put forth was that moderate GR activity is optimal for cognitive processes, but too much GR activity, such as that which occurs following stress, has negative repercussions for synaptic plasticity and, therefore, learning. Support for this idea came from studies reporting a curvilinear, U-shaped relationship between corticosteroids and hippocampal synaptic plasticity and learning (Diamond et al., 1992; Andreano and Cahill, 2006), as well as from research showing that extensive GR activity results in excessive calcium influx and negative gene-dependent effects on cellular function (Joels et al., 2003). Combined with work on chronic stress and corticosteroid-hippocampal volume relationships observed in humans with psychological disorders (Campbell et al., 2004; Zoladz and Diamond, 2013), a majority of the research led investigators to conclude that stress generally exerts deleterious effects on hippocampal structure and function.

Over time, a greater appreciation for the complexity of stress-memory interactions arose, as evidence accumulated suggesting that stress could enhance, impair or have no effect on hippocampus-dependent learning and synaptic plasticity. Researchers began showing that corticosteroids not only have delayed, gene-dependent, negative consequences on cellular activity, but can also exert rapid, non-genomic, facilitative effects (Orchinik et al., 1991; Karst et al., 2005). This led to much different theoretical approaches to how stress affects cognition, including an appreciation for the timing of the stress relative to learning or memory, the sex of the organism being investigated, and the type of learning and memory being assessed, to name a few (Joels et al., 2006; Diamond et al., 2007; Wolf, 2009; Joels et al., 2011; Schwabe et al., 2012). Diamond and colleagues, echoing prior theoretical views (Diamond et al., 1990; Shors and Thompson, 1992), put forth another idea – that stress might impair memory by producing a memory of its own (Diamond et al., 2004, 2005). Here, we have extended this view to consider how stress, as a memory formation process, time-dependently affects encoding, consolidation, and retrieval.

## Stress as a Learning Event

For the past several decades, long-term potentiation (LTP) has been studied as a putative physiological mechanism underlying memory formation (Shors and Matzel, 1997; Kim and Yoon, 1998; Diamond et al., 2007; Joels and Krugers, 2007). LTP is a long-lasting enhancement of synaptic efficacy that results from high-frequency stimulation (HFS) of afferent fibers (Hebb, 1949; Marr, 1971; Lomo, 2003) and can be performed *in vitro* (in brain slices), in awake and behaving animals, or in anesthetized animals (Lynch, 2004). *In vitro* setups keep brain tissue functional via artificial cerebrospinal fluid and allow investigators to stimulate and record from populations of neurons. Setups in awake or anesthetized animals involve intracerebral implantation of stimulating and recording electrodes via stereotaxic surgery; these electrodes can subsequently be used to examine LTP induction. Successful memory formation for a learning event is believed to coincide with the strengthening of neural connections and a lasting pattern of altered synaptic weights. However, if multiple LTP-inducing events occur in close proximity, the limited number of available neurons may result in a “ruthless competition” for access to synaptic plasticity production and successful memory formation (Diamond et al., 2004, 2005). In other words, with limited resources, the brain would be forced to prioritize information that is more important.

In an effort to understand the dynamic nature of hippocampus-dependent memory formation, researchers have examined the influence of LTP induction on subsequent hippocampal synaptic plasticity and learning. Application of HFS to afferent fibers has been shown to produce widespread saturation of hippocampal synapses, and the long-lasting alteration of synaptic weights produced by this HFS can lead to an inhibition of subsequent LTP and hippocampus-dependent learning (Huang et al., 1992; Barnes et al., 1994; Moser and Moser, 1998, 1999; Otnaess et al., 1999). This activity-dependent modification of synaptic efficacy has been termed metaplasticity, corresponding to the notion that a prior change in synaptic plasticity can influence the direction and degree of subsequent changes in synaptic plasticity (Abraham and Bear, 1996).

Because we know that prior LTP induction can influence subsequent LTP induction, it stands to reason that the formation of one memory could influence subsequent memory formation. However, research has shown that this tends to occur only when a learning task produces widespread synaptic saturation (extensively reviewed in Diamond et al., 2004). Learning events that produce such a strong memory or change in synaptic plasticity are those that have a strong emotional component and elicit a significant stress response. Accordingly, research has revealed very similar molecular mechanisms underlying stress- and LTP-induced changes in hippocampal function (see Huang et al., 2005 for a review). Some of these commonalities include increased early gene induction (Cole et al., 1989; Schreiber et al., 1991; Platenik et al., 2000), increased NMDA and AMPA receptor activity (Tocco et al., 1991, 1992; Kim et al., 1996; Brun et al., 2001), increased levels of neurotrophins [e.g., brain-derived neurotropic factor (BDNF; Gooney and Lynch, 2001; Marmigere et al., 2003)] and increased glutamate and intracellular calcium levels (Sapolsky, 1996; Abraham et al., 1998; Hossmann, 1999; Venero and Borrell, 1999; Joels, 2001; Takahashi et al., 2002; Joels et al., 2003; McEwen and Chattarji, 2004). Additional evidence for shared mechanisms between artificially induced LTP and stress-induced neuroplasticity is research indicating that NMDA receptor antagonists, which impair LTP induction, prevent the effects of stress on subsequent hippocampus-dependent learning and LTP (Kim et al., 1996; Park et al., 2004). In theory, the NMDA receptor antagonists block the stress-memory formation, which allows subsequent hippocampus-dependent learning and LTP to occur. Research has also shown that despite stress impairing subsequent learning and LTP induction, the memory for the stress-inducing event remains intact (Diamond et al., 2004; Zoladz et al., 2010). Together, these findings have provided support for the idea that stress induces an endogenous form of LTP that allows a memory of the stress experience to be formed. Although this is adaptive, because it allows an organism to remember the stress experience, in some cases it can also serve to impair subsequent cognitive processing.

## Stress Effects on Learning and Memory and the Important Role of Timing

Knowing that stress exposure results in the activation of molecular mechanisms that are remarkably similar to those observed as a result of artificially induced synaptic plasticity, we might consider stress, itself, as a memory-producing event. Viewed in this light, stress, and the waves of psychobiological responses that result from such a learning event, can be expected to strongly influence the successful encoding, consolidation, and retrieval of unrelated information (i.e., information not related to the stressor).

Stress effects on the retrieval of previously learned information could be understood as the formation of one memory (i.e., the stress-memory) interfering with the retrieval of another memory. Similar to this line of reasoning, studies have shown that LTP can produce retrograde amnesia for previously learned information (McNaughton et al., 1986; Brun et al., 2001). In theory, the initial learning task (spatial learning in this case) leads to the potentiation of a small subset of synapses, and the pre-retrieval LTP leads to a complete saturation of synaptic nodes. The all-encompassing wave of plasticity that results from the LTP induction alters the pattern of synaptic weights throughout the hippocampus, resulting in an impaired ability to retrieve the previously formed memory. Consistent with these findings, and as described above, studies examining stress effects on retrieval have found that acute stress exposure that occurs before a memory test leads to impaired memory performance, while preserving the memory that has formed as a result of stress exposure (Diamond et al., 2004; Zoladz et al., 2010). In the referenced studies, rats trained in an inhibitory avoidance task that involved foot shock as an unconditioned stimulus (i.e., a stressor) exhibited impaired spatial memory retrieval, despite retaining the shock-induced fear memory that was formed in the inhibitory avoidance task. This effect was observed when the spatial learning and memory occurred on the same day or when they were separated by 24 h. Importantly, the memory impairment may not result from complete elimination of the original memory. Instead, the stress-induced neuroplasticity may cause an impaired ability to activate the synapses required to retrieve the previously formed memory (Diamond et al., 2004).

As described above, post-learning stress almost always enhances long-term memory, but pre-learning stress effects on long-term memory have resulted in inconsistent findings. The timing of stress relative to learning has been shown to influence both types of effects. Studies in which learning or HFS of afferent fibers occurred immediately before or after stress exposure revealed a significant enhancement of long-term memory or an increase in the duration of hippocampal LTP (reviewed in Diamond et al., 2007). In the same way that stress exposure immediately after a learning event leads to a strong memory formation for both the stress event and unrelated learning event, acute stress exposure occurring immediately before an unrelated learning event typically facilitates memory formation for both events (note that the facilitation can be selective for emotional/neutral or central/peripheral information). Alternatively, when acute stress exposure is temporally separated from a prior or subsequent unrelated learning event, memory formation for that learning event is often impaired (pre-learning stress) or unaffected (post-learning stress).

**FIGURE 1:**
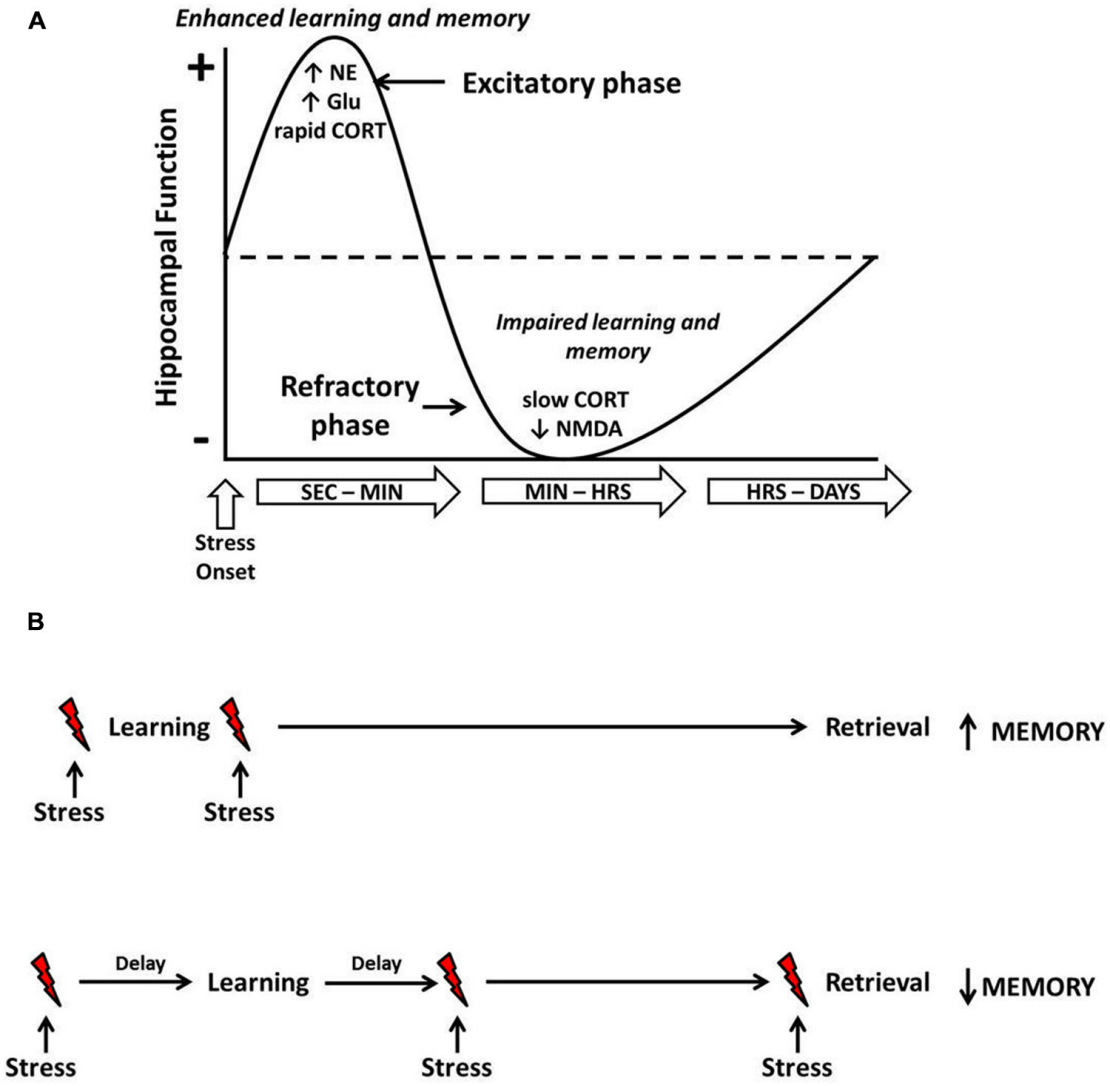
**Temporal dynamics of acute stress effects on hippocampus-dependent learning and memory**. Shortly following onset, stress induces a rapid increase in norepinephrine (NE), glutamate (Glu) and several other neurochemical substances (e.g., CRH, acetylcholine, dopamine, etc.). Within a few minutes, corticosteroids (CORT) are also released and can exert rapid, non-genomic effects on cellular activity. Combined, this rapid stress-induced neurochemical activity results in an enhancement of hippocampal function, and learning that occurs around this time frame would be enhanced **(A)**. However, as time and/or the stressor continues, desensitization of glutamatergic NMDA receptors and delayed, gene-dependent corticosteroid activity results in an inhibition of hippocampal function, and learning that occurs around this time frame would be impaired. The bottom figure **(B)** illustrates these principles. When stress (indicated by the red lightning bolts) occurs in close temporal proximity to learning, long-term memory retrieval will be enhanced. When the stressor is temporally separated from the learning or occurs prior to retrieval, long-term memory will be impaired.

Based on the seemingly time-dependent effects of stress on hippocampal function, Diamond et al. (2007) developed the temporal dynamics model of emotional memory processing. This model illustrates the biphasic modulation of hippocampal function by stress-induced amygdala activity and is described in **Figure 1**. The first phase encompasses a rapid enhancement of hippocampal plasticity resulting from stress-induced neurochemical interactions in the amygdala and hippocampus. Support for this phase comes from electrophysiological work showing that stimulation of the amygdala immediately prior to HFS in the hippocampus results in strengthened synaptic connections in the hippocampus (Akirav and Richter-Levin, 1999, 2002). Importantly, this amygdala-induced enhancement of hippocampal plasticity depends on both noradrenergic and corticosteroid mechanisms. During this phase, corticosteroids released as a result of stress would be expected to exert rapid, excitatory effects on hippocampal function as a result of non-genomic activity (Karst et al., 2005; Karst and Joels, 2005). The stress-induced facilitation of hippocampal function, however, is short lived and may last only minutes after onset of the stress experience. The second phase of the model represents a refractory period during which the acquisition of new information or LTP induction would be improbable. This refractory period is caused by the desensitization of glutamatergic receptors, which have been over-stimulated by stress-induced glutamate release, and delayed, gene-dependent activity of corticosteroids. Accordingly, in electrophysiological work, when amygdala stimulation and hippocampal HFS are separated in time, the resulting synaptic change is a suppression of hippocampal LTP. Thus, application of tetanic stimulation during the refractory period would likely fail to overcome the newly elevated threshold for LTP induction.

Consistent with the temporal dynamics model, Schwabe et al. (2012) proposed that the rapid stress-induced increase of catecholamine and non-genomic corticosteroid activity puts an organism in a ‘memory formation mode,’ which results in enhanced memory production for a stressful event and information that is temporally proximal to such an event. However, as the stress continues and/or upon the initiation of gene-dependent corticosteroid activity, a ‘memory storage mode’ is induced, which impairs cognitive processes that could compete or interfere with the storage of information about the stress event. Both Diamond and Schwabe would likely agree that the temporal dynamics of memory processing subsequent to stress exposure is adaptive, despite the fact that it can result in enhancing *or* deleterious effects on learning and memory. When a stressful experience occurs, it is beneficial to survival for an organism to form a strong memory of that event. Moreover, the suppression of subsequent cognitive processing or memory formation would also be advantageous because it allows the brain to focus on storing the stress-related memory, without interference from competing cognitive processes.

The temporal dynamics model addresses the functionality of the hippocampus from stress onset to hours after stress exposure. This information can be extended to understand how stress, as a learning experience, influences additional unrelated learning experiences. We have summarized the research studies that have examined the effects of acute stress, administered at different time points, on hippocampus-dependent learning and memory in **Table 1**. Stress-induced facilitation or impairment of unrelated information will be largely determined by the convergence of information from the stress and learning experiences in “time” and “space” (Joels et al., 2006). Convergence of the experiences in “time” relates to the idea of stress and learning occurring in close temporal proximity, whereas the convergence in “space” refers to the two events sharing mutual brain circuits that overlap during the memory formation process. As such, stress will enhance memory when a learning event occurs in the same context as the stress event, or when the two events occur closely in time. This enhancement results from shared neural circuits simultaneously forming memories for the learning event and stress event. As memory formation for a stress event is characterized by rapid psychobiological responses that allow for strong memory development, the resulting alteration of synaptic plasticity encodes information for both learning experiences. This enhanced consolidation for arousing experiences and learning events that occur in close proximity is adaptive in nature, allotting the highest priority of memory formation to events that code for information relevant to survival.

Alternatively, if a learning event occurs outside of a stress event context, or the events are temporally separated, encoding for this unrelated information would be significantly impaired as the neural circuits necessary for memory formation had already been previously saturated. As discussed, the hippocampus descends into a refractory period shortly following stress onset due to stress-induced synaptic saturation. Although the hippocampus does not display complete suppression, memory formation will be severely impaired. Much like the rapid facilitation of hippocampal LTP serves adaptive purposes, the refractory period also offers the organism benefits. The first benefit is a protection against increased glutamate exposure, which would eventually lead to neurotoxicity; second, the refractory period offers a short window in time in which the emotional memory experience can reduce corruption by subsequent learning; third, it allows for the consolidation of emotional information acquired in phase one (Diamond et al., 2007).

## Conclusion and Caveats

Initial support for the temporal dynamics model of emotional memory processing came from preclinical work showing that a brief stressor applied immediately before learning could enhance long-term spatial memory in rats (Diamond et al., 2007). If the stressor was separated from the learning by a period of 30 min, however, no memory enhancement was observed. More recently, investigators have extended the work to humans. This research has, for the most part, provided much needed support for the temporal dynamics model in people (Zoladz et al., 2011a, 2013, 2014b,c; Quaedflieg et al., 2013). However, some issues have arisen. One is that the sex of the organism appears to be influential in the types of effects that stressor timing has on learning and memory. Indeed, the temporal dynamics model was originally based on research performed in male, but not female, rodents. Thus, it is perhaps not surprising that work in humans has shown that females can respond very differently to the same stressor. As an example, Zoladz et al. (2013) reported that males, but not females, exhibited an impairment of long-term memory when exposed to a brief stressor 30 min prior to learning. These investigators also showed that stress immediately before learning reduced false memory production in males and females but enhanced true memory in females only (Zoladz et al., 2014c).

One factor that may underlie these observed sex-dependent effects is the modulatory role female sex hormones can exert on physiological mechanisms involved in memory formation. As many studies that have included female participants did not control for phase of the menstrual cycle, levels of estrogen and progesterone, or use of oral contraceptives, the possible interaction that may be occurring between female sex hormones and the time-dependent effects of stress-induced neurochemicals is not well understood. Further research investigating the modulatory role that female sex hormones may be playing in stress effects on learning and memory may offer much needed information as to how the timing of stress differentially influences learning and memory in males versus females.

An additional nuance that is important when considering the temporal dynamics model is the severity of the stressor. For severe stressors, the excitation phase of hippocampal function could be much more short-lived, or in the case of traumatic stressors, non-existent. For milder stressors, it is possible that the excitation phase could last longer. That the temporal dynamics of stress-induced alterations of hippocampal function could vary from stressor to stressor could relate to individual differences in physiological responses to stress, perceptions of control in times of stress, and what constitutes a stressful event. Numerous studies have shown that some individuals respond strongly to laboratory stressors (defined as “Responders”), while others show little changes in SNS or HPA axis activity (defined as “Non-Responders”). Moreover, it may be useful to consider what type of genetic variations across individuals could make them more or less susceptible to stress-induced changes in amygdala and hippocampal function. For instance, in a recent study, we showed that female carriers of the ADRA2B deletion variant (a genetic alteration that make the noradrenergic system more responsive to stress) were more susceptible to stress-induced enhancements of long-term memory (Zoladz et al., 2014b). If some genetic variants influence susceptibility to stress-induced enhancements of long-term memory, this could lend insight into who is more likely to form an intrusive, traumatic memory following extreme stress.

Finally, it is worth noting that the idea of stress inducing an amygdala-dependent biphasic effect on hippocampal function is largely dependent on electrophysiological work focusing more exclusively on the perforant pathway, which terminates in the dentate gyrus of the hippocampus. Other electrophysiological work has shown that corticosteorids can exert much different effects on different hippocampal subregions, such as CA1 and CA3 (Joels et al., 2009). Therefore, stress-induced amygdala activity, which biphasically influences dentate gyrus LTP, could affect other areas of the hippocampus in a different time-dependent manner.

Clearly, stress can exert differential effects on learning and memory depending on when it is administered and how long it lasts. Although the temporal dynamics notion may be an oversimplification of an overly complex area of research, it provides a useful guide for understanding how stress time-dependently influences learning and its neurobiological basis. Future work is necessary to clarify how timing interacts with stress effects on memory and how sex and individual differences can influence these effects.

## Conflict of Interest Statement

The authors declare that the research was conducted in the absence of any commercial or financial relationships that could be construed as a potential conflict of interest.
